# Mapping QTL for Plant Architecture-Related Traits in Soybean Across Multiple Environments

**DOI:** 10.3390/plants15132005

**Published:** 2026-06-28

**Authors:** Tao Wang, Qiang Chen, Xu Wang, Long Yan, Xiao-Lei Shi, Xiao-Dong Tang, Xiao-Tong Lei, Fu-Ming Xiao, Meng-Chen Zhang

**Affiliations:** 1 Hebei Laboratory of Crop Genetics and Breeding, National Soybean Improvement Center Shijiazhuang Sub-Center, Huang-Huai-Hai Key Laboratory of Biology and Genetic Improvement of Soybean, Ministry of Agriculture and Rural Affairs, Institute of Cereal and Oil Crops, Hebei Academy of Agricultural and Forestry Sciences, Shijiazhuang 050035, China; 2 Handan Academy of Agricultural Sciences, Handan 056001, China

**Keywords:** soybean, plant architecture, quantitative trait loci, QE interaction effect

## Abstract

Improving soybean plant architecture is critical for enhancing yield potential. To dissect the genetics of related traits, a recombinant inbred line population of 175 F_9_:_12_ families (derived from Glycine max cultivars Jidou 12 [female] × Ji NF58 [male]) was used for quantitative trait locus (QTL) mapping. Four key traits—plant height, bottom pod height, node number on main stem, and branch number—were analyzed across six environments (two growing seasons × three locations) via two methods: composite interval mapping (CIM, QTL Cartographer v2.5) and mixed-model-based composite interval mapping (MCIM, QTLNetwork 2.0). A total of 22 stable QTLs were detected, with phenotypic variation explained (PVE) of 1.2–52.5%. Co-localized QTLs (due to significant trait correlations) concentrated in three genomic intervals: Satt286-Sat_251 (LG C2/chromosome 06), Satt156-Satt229 (LG L/chromosome 19), and Satt581-Sat_190 (LG O/chromosome 10). A novel QTL (*qBPH-O-2*) for bottom pod height was identified on LG O. Major QTLs with QTL-by-environment (QE) interactions were found on LG A1 (plant height, node number on main stem) and *qBN-C2-1* (branch number, high additive effects + QE interactions). These findings support marker-assisted selection (MAS), targeted plant architecture improvement, and gene pyramiding in soybean breeding.

## 1. Introduction

Soybean is a dual-purpose crop for oil and grain, serving as a major source of plant protein and oil for humans [1,2,3] (Nadeem et al. 2021, Liu et al. 2018, Jang et al. 2015). With the upgrading of consumption structures, the global demand for soybean has been increasing year by year, and soybean plant architecture traits play a crucial role in yield formation [4,5]. Plant architecture is a core agronomic trait that affects crop light energy use efficiency, stress resistance, and final yield. Optimizing plant architecture is a key goal of crop breeding, which has achieved remarkable results in the “Green Revolution” of crops such as rice and wheat. In soybeans, rational plant architecture is an important breakthrough to overcome yield bottlenecks.

In rice and wheat, semi-dwarf varieties have enabled significant yield increases. Semi-dwarf genes (e.g., wheat *Rht* and rice *Sd1*) regulate gibberellin synthesis and signaling to reduce plant height, enhance lodging resistance, improve fertilizer use efficiency, and adapt to high-density planting, avoiding yield losses from excessive height-related lodging [6,7]. In maize, compact leaf angles reduce shading under high density, boosting light capture and grain yield per unit area [8]. For soybean, plant architecture optimization is more complex but vital.

With the development of molecular markers, especially the emergence of SSR, SNP, and Indel markers, QTLs associated with soybean plant architecture traits have been gradually mapped to corresponding linkage groups. The SoyBase database (https://www.soybase.org) has documented numerous QTLs related to plant architecture traits, which are widely distributed across 18 linkage groups, with a higher concentration on groups C2 and L. These two groups are speculated to be QTL-rich regions controlling plant height and node number on main stem, where major-effect QTLs may stably exist. Specht et al. [9], Lark et al. [10], Mansur et al. [11], Lee et al. [12], Zhang et al. [13], Cao et al. [14], and Fang et al. [15] have all mapped QTLs related to plant height or main stem node number on these two groups. There are also several reports on QTL mapping for branch number. Sayama et al. [16] detected five QTLs controlling branch number on groups B1, C2, D2, L, and G based on an F9 recombinant inbred line (RIL) population, which explained 3.0–15.7% of phenotypic variation; Shim et al. [17] constructed a high-resolution genetic map using the BARCSoySNP6K chip and identified a major QTL for branch number on chromosome 06 with an R^2^ of 14.5%. As an important plant architecture trait, bottom pod height is a key characteristic for evaluating soybean varieties suitable for mechanical harvesting, and it provides guidance for soybean mechanical harvesting operations [18]. According to the research results of Ramteke et al. [19], the bottom pod height should be at least 12 cm to avoid grain loss during mechanical harvesting. Liang et al. [20] used 447 recombinant inbred lines to detect QTLs related to major agronomic traits and identified five QTLs highly correlated with bottom pod height on four linkage groups, namely A2, C2, J_1, and J_2. Jiang et al. [18] used 147 recombinant inbred lines derived from a cross between “Dongnong594” and “Charleston” over 8 years to identify the major QTLs associated with bottom pod height. Using the composite interval mapping method with WinQTLCart (version 2.5), 11 major QTLs were identified, which were located on linkage groups L, D1b, D2, L, M, and J, respectively. The R^2^ (the contribution rate of the QTL) for all of the QTLs ranged from 6.50 to 15.30%. Lou et al. [21] constructed a high-density genetic linkage map using genotyping-by-sequencing (GBS) of an F_3_ population and identified 19 QTLs related to bottom pod height on chromosomes 1L, 1S, 2, 3, 5, and 6. By annotating the QTL *qFPH6-1* interval, 36 genes that may be related to bottom pod height were identified. Despite numerous reports on QTLs related to soybean plant architecture traits, results vary due to differences in experimental materials, environments, conditions, and methods. Additionally, quantitative traits are susceptible to environmental influences, leading to the inability to replicate and validate many mapped QTLs. Only QTLs stably expressed across multiple environments and years can provide practical value for crop breeding.

In this study, a high-generation RIL population consisting of 175 families was used as experimental material. Correlation analysis was performed for four plant architecture traits (plant height, bottom pod height, branch number, and node number on main stem) across six environments (two years and three locations). Using a constructed genetic linkage map and genetic statistical software with two algorithms, QTL mapping was conducted to identify stable major-effect QTLs across different environments and clarify genotype–environment interaction effects. This study provides a molecular theoretical basis for the improvement of soybean plant architecture traits.

## 2. Materials and Methods

### 2.1. Plant Materials

A F_12_ RIL population consisting of 175 families was developed from a cross between Jidou 12 (female parent) and Ji NF58 (male parent). Jidou 12 is a high-protein soybean cultivar bred by the Institute of Cereal and Oil Crops, Hebei Academy of Agriculture and Forestry Sciences, China. It exhibits a determinate growth habit, dwarf plant stature, numerous branches, a pyramidal plant architecture, and elliptical seeds with a 100-seed weight of 23–27 g, and belongs to the large-seed type. Ji NF58 is a high-oil soybean cultivar with a semi-determinate growth habit, tall plant stature, few branches, round seeds with a 100-seed weight of 14–16 g, and is classified as the small-seed type.

### 2.2. Field Design and Trait Investigation

The RIL population and their parental lines were planted at three experimental locations (Shijiazhuang, Cangzhou, and Handan) over the course of two years (Y1 and Y2). The field experiment was arranged in a randomized complete block design with three replications. Each plot consisted of three rows, with a row length of 2.0 m, row spacing of 0.5 m, and hill spacing of 0.1 m. Conventional field management practices were applied throughout the growing season.

At maturity, ten consecutive plants from the middle row of each plot were selected for trait measurement. Plant height (PH) was measured as the distance from the soil surface to the apical meristem of the main stem. Bottom pod height (BPH) was measured as the distance from the soil surface to the lowest pod-bearing node on the main stem. Branch number (BN) was recorded as the total number of effective branches (branches bearing at least one pod) on the main stem. Node number on main stem (NNMS) was counted as the total number of nodes on the main stem from the cotyledonary node to the apical node.

### 2.3. SSR Primer Screening

A total of 347 pairs of SSR primers were selected evenly across 20 soybean linkage groups based on Cregan’s soybean consensus map. Among these, there were 134 pairs of polymorphic SSR primers between the two parents. After filtering for segregation distortion, low amplification quality, ambiguous banding patterns, and excessive missing data, 117 high-quality polymorphic SSR markers were retained and used for genotyping and genetic linkage map construction.

### 2.4. DNA Extraction, PCR Amplification, and Detection of Amplified Products

Equal amounts of fresh leaves were randomly sampled from each RIL and parental line. Genomic DNA was extracted using the SDS method.

The PCR amplification was performed in a 20 µL reaction volume containing 20 ng of total DNA, 0.3 µL of each forward and reverse primer, 1.5 µL of dNTPs, 2.0 µL of 10 × PCR buffer, 0.2 µL of Taq polymerase, and ddH_2_O to make up the final volume. The PCR cycling conditions were as follows: initial denaturation at 94 °C for 5 min; followed by 35 cycles of denaturation at 94 °C for 30 s, annealing at 47 °C for 30 s, and extension at 72 °C for 30 s; and a final extension at 72 °C for 5 min. The PCR products were stored at 4 °C until further analysis.

Denaturing polyacrylamide gel electrophoresis (PAGE) was used for product separation. PCR samples were denatured at 95 °C for 3 min and immediately cooled on ice. The denatured products were separated on 6% polyacrylamide sequencing gels at a constant power of 90 W for approximately 90 min (adjusted according to fragment size). Gels were stained using the silver staining method, and images were captured after development. SSR marker genotypes identical to Jidou 12 were scored as “A”, those identical to Ji NF58 as “B”, heterozygous genotypes as “H”, and missing or ambiguous genotypes as “_”.

### 2.5. Correlation Analysis

Phenotypic correlations among traits were analyzed using SPSS Statistics 17.0 software. A genetic linkage map of the RIL population was constructed with Map Manager QTXb20 (April 2004) software, and recombination frequencies were converted into map distances (centimorgans, cM) using the Kosambi mapping function.

### 2.6. QTL Mapping

Joint QTL mapping across multiple environments was performed using composite interval mapping (CIM) in QTL Cartographer v2.5 and mixed-model-based composite interval mapping (MCIM) in QTLNetwork 2.0. This combined approach improved the accuracy of QTL detection and avoided missing major-effect QTLs. The window size was set to 10 cM for both methods, with a walk speed of 2 cM and 1 cM for CIM and MCIM, respectively. For composite interval mapping (CIM), the LOD threshold was determined by 1000 permutation tests at the experiment-wise significance level of *p* < 0.05. For mixed-model-based composite interval mapping (MCIM) in QTLNetwork 2.0, the critical F-value threshold was determined by the *p* = 0.05 significance level to declare significant QTLs and QTL-by-environment interaction effects. Only significant QTLs were reported in this study. QTLs for the same trait with LOD peaks separated by less than 5 cM were considered a single QTL. The QTL naming was based on the method of McCouch [22].

The following parameters were used to evaluate QTLs: F score (significance), additive effect (direction of genetic effect), H^2^A (%) (phenotypic variation explained by additive effects), and H^2^AEi (%) (phenotypic variation explained by QTL × environment interactions).

## 3. Results

### 3.1. Phenotypic Characteristics of Plant Architecture Traits

**Figure 1:**
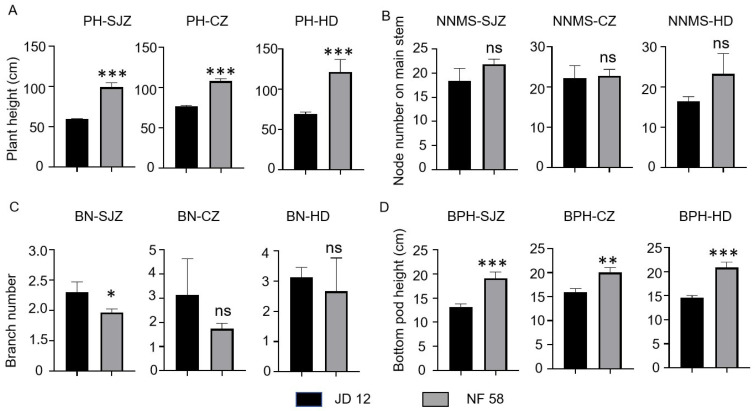
Phenotypic comparison of four plant architecture traits between the two parental lines. (**A**) Plant height; (**B**) node number on main stem; (**C**) branch number; (**D**) bottom pod height. Values are means ± standard error (SE). * Significant difference at *p* < 0.05, ** significant difference at *p* < 0.01, *** significant difference at *p* < 0.001 (*t*-test), ns, not significant. JD12: Jidou 12; NF58: Ji NF58.

Significant phenotypic differences were observed between the two parents for all four traits (Figure 1). Jidou 12 exhibited significantly lower plant height, bottom pod height, and node number on main stem, but higher branch number than Ji NF58 (*p* < 0.01). These stable and contrasting phenotypic differences provide an ideal genetic basis for QTL mapping in the RIL population.

Transgressive segregation was observed for all plant architecture traits in the RIL population across all six environments, indicating sufficient genetic variation for subsequent quantitative trait locus (QTL) mapping. Among the four traits, plant height exhibited the largest standard deviation, reflecting high phenotypic divergence and significant differences among RIL families. Plant height ranged from 17.53 cm to 195.17 cm, with kurtosis and skewness values ranging from −0.98 to −1.14 and 0.14 to 0.47, respectively. In contrast, branch number showed the lowest phenotypic variation, with a range of 0 to 8, and kurtosis and skewness values of −0.47 to 1.86 and 0.56 to 1.51, respectively.

For bottom pod height, the phenotypic range was 5.33 cm to 46.17 cm, with kurtosis and skewness ranging from 0.42 to 1.95 and 0.41 to 1.21, respectively. The number of main stem nodes varied from 6.27 to 32.50, with kurtosis and skewness values of −1.37 to 0.54 and −0.09 to 0.72, respectively. Notably, the absolute values of kurtosis and skewness for all traits were less than 1 in most environments, consistent with a normal distribution (Table 1)—a critical prerequisite for valid QTL mapping analysis.

Broad-sense heritability (H^2^) ranged from 66.9% to 88.1% across environments, with higher heritability for plant height and node number on main stem(83.7–88.1%) and moderate heritability for branch number (66.9–72.5%), indicating strong genetic control and moderate environmental influence, consistent with the observed QTL × environment interactions.

### 3.2. Correlation Analysis Among Plant Architecture Traits

Correlation analysis across six environments revealed consistent patterns for most trait pairs (Table 2).

Specifically, PH, BPH, and NNMS showed highly significant positive correlations (*p* < 0.01) with each other, with correlation coefficients ranging from 0.69 to 0.95. This suggests a strong genetic linkage or pleiotropy among these three traits, which may coordinate to shape soybean plant architecture.

In contrast, the correlation of branch number with the other three traits was environment-dependent: significant correlations (*p* < 0.05 or *p* < 0.01) were detected in Shijiazhuang and Cangzhou, but no significant correlation was observed in Handan. This result implies that environmental factors (e.g., soil fertility, temperature, or precipitation) may exert a greater influence on the genetic regulation of branch number compared to the other three traits.

### 3.3. QTL Mapping and QTL-by-Environment (QE) Interaction Analysis for Plant Architecture Traits

**Figure 2:**
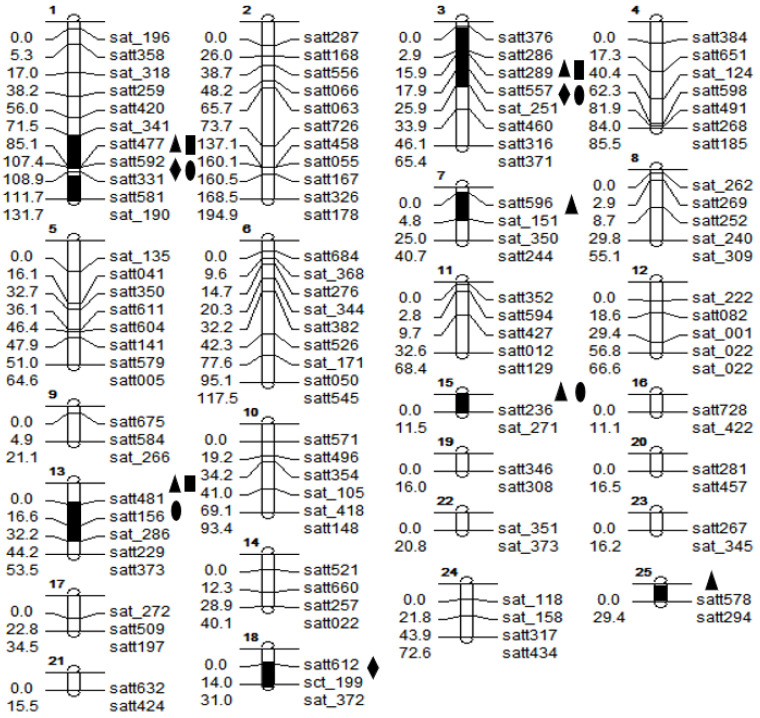
Genetic linkage map of the RIL population and QTLs positions for four plant architecture traits detected by CIM and MCIM. 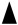: QTLs of plant height; 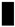: QTLs of bottom pod height; 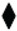: QTLs of branch number; 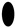: QTLs of node number on main stem. The correspondence between the linkage groups in the figure and the public map: 1-O LG; 3-C_2_ LG; 7-J LG; 10-I LG; 13-L LG; 15-A_1_ LG; 18-G LG; 25-C_1_ LG. CIM: composite interval mapping of WinQTLCart v2.5; MCIM: the mixed linear model interval mapping of Network2.0.

A genetic linkage map was constructed using 117 polymorphic simple sequence repeat (SSR) markers, spanning a total genetic distance of 1373.6 cM. The markers were distributed across 20 soybean linkage groups (LGs), with each LG containing 2 to 11 markers and covering a genetic distance of 11.10 to 194.90 cM (Figure 2). The average marker density of the present genetic map was comparable to that of previously published soybean linkage maps. Markers were evenly distributed across all 20 soybean linkage groups without large gaps, achieving full genome coverage of soybean chromosomes. Such marker saturation is adequate to capture both major-effect QTLs and most minor-effect QTLs with low phenotypic contribution in subsequent CIM analysis, which guarantees robust and credible QTL identification results [23].

**Figure 3:**
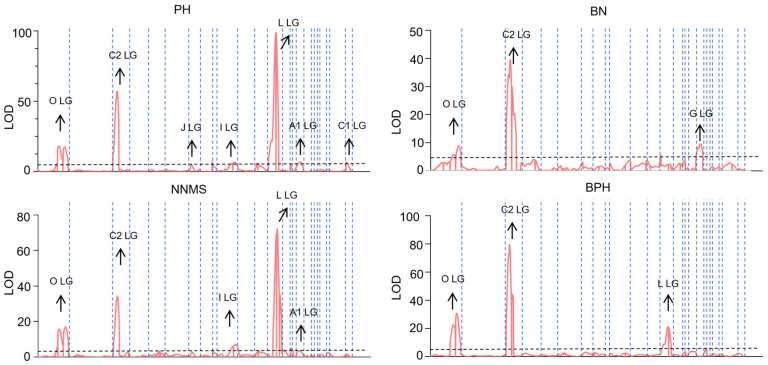
Test statistics of genome scan for QTLs of four plant type traits in MCIM. PH: plant height; BPH: bottom pod height; BN: branch number; NNMS: node number on main stem.

A total of 22 QTLs associated with plant architecture traits were detected across six environments. Among these, 16 QTLs were identified using composite interval mapping (CIM) with PVE ranging from 5.40% to 52.50% (Table 3), and 20 QTLs were detected using mixed-model-based composite interval mapping (MCIM) with PVE of 1.20% to 24.80% (Figure 3 and Table 4).

**Figure 4:**
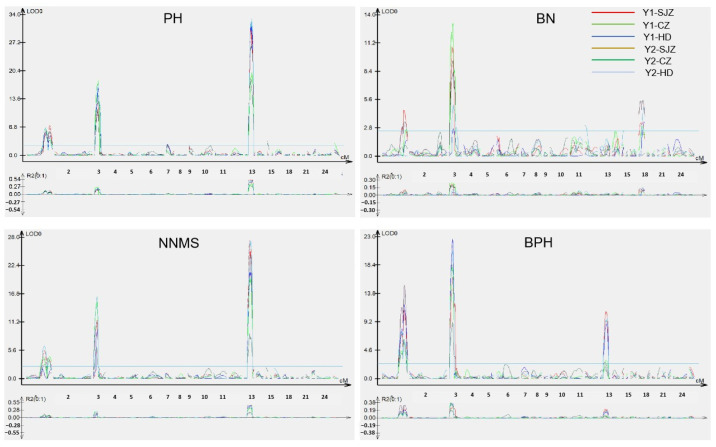
Likelihood ratio profiles of QTLs of four plant architecture traits across six environments detected by CIM. PH: plant height; BPH: bottom pod height; BN: branch number; NNMS: node number on main stem.

Eight QTLs controlling plant height were mapped to linkage groups A1, C1, C2, I, J, L, and O. Among them, four QTLs (*qPH-C2-1* on linkage group C2, *qPH-L-1* on linkage group L, *qPH-O-1* and *qPH-O-2* on linkage group O) were stably detected across six environments and two mapping models (Figure 4). *qPH-C2-1* was located in the Satt286-Sat_251 interval, with the favorable allele derived from Jidou 12, explaining 11.7% to 27.2% of the phenotypic variation. *qPH-L-1* was positioned in the Satt156-Satt229 interval, with the favorable allele from Jinong NF58, accounting for 24.8% to 52.5% of the phenotypic variation. *qPH-O-1* and *qPH-O-2* were located in the Satt477-Satt592 and Satt581-Sat_190 intervals, respectively, with favorable alleles from Jinong NF58 and PVE of 4.00% to 14.30%. All eight QTLs showed QE interaction effects to varying degrees, with the PVE of individual QE interactions ranging from 0.10% to 0.50%. Only *qPH-A1-1* exhibited a higher PVE for QE interaction than for additive effect.

Six QTLs associated with the number of main stem nodes were mapped to LGs A1, C2, I, L, and O. The QTLs on LGs C2, L, and O (e.g., *qNNMS-C2-1*, *qNNMS*-*L-1*) were stably detected across environments and mapping methods, and their marker intervals overlapped with those of plant height QTLs on the same LGs. This co-localization supports the significant positive correlation between plant height and node number on the main stem (Section 3.2). The PVE of these QTLs ranged from 3.60% to 52.30%. All QTLs showed QE interaction effects (PVE = 0.1–0.5%), and except for *qNNMS*-*A1-1*, all other QTLs had higher additive effect PVE than QE interaction PVE.

Four stable QTLs for bottom pod height were mapped to LGs C2, L, and O, with marker intervals overlapping with plant height QTLs on the corresponding LGs (consistent with their phenotypic correlation). Key QTLs included: *qBPH-C2-1* (LG C2): favorable allele from Jidou 12, PVE = 19.60–36.00%. *qBPH-L-1* (LG L) and *qBPH-O-2* (LG O): favorable alleles from Jinong NF58, PVE = 8.10–20.70% and 6.90–31.70%, respectively. The QE interaction PVE for individual QTLs ranged from 0.30% to 1.30%, all lower than the additive effect PVE, indicating that these QTLs are genetically stable.

Four stable QTLs associated with branch number were stably detected across multiple environments, mapped to linkage groups C2, G, and O. Among these, *qBN-C2-1* and *qBN-O-2* overlapped with the intervals of plant height QTLs on the corresponding linkage groups. *qBN-C2-1* was detected in all six environments, with the favorable allele from Jidou 12, explaining 6.50% to 28.30% of the phenotypic variation. *qBN-O-2* was only detected in Shijiazhuang and Cangzhou, with the favorable allele from Jinong NF58 and PVE of 1.20% to 8.40%. *qBN-G-1* was located in the Sct_199-Sat_372 interval on linkage group G, with the favorable allele from Jinong NF58, accounting for 2.60% to 14.70% of the phenotypic variation. Notably, *qBN-C2-1* exhibited the highest QE interaction effect, with a PVE of 2.80% for the QE effect.

## 4. Discussion

Soybean plant architecture traits are quantitative traits controlled by multiple genes [24]. Numerous QTL mapping studies on plant architecture have been published, whereas few studies implement multi-year and multi-location joint QTL analysis with two complementary mapping algorithms. Joint multi-environment analysis improves QTL detection reliability and optimizes the estimation of QTL location and genetic effect [25]. Moreover, QTL repeatedly validated via CIM and MCIM substantially enhance mapping credibility and reduce the omission probability of major-effect loci.

The stable QTLs identified in this study were clustered within four core genomic regions: Satt286-Sat_251 (C2), Satt156-Satt229 (L), Satt477-Satt592 (O) and Satt581-Sat_190 (O). Consistent co-localization of PH, BPH and NNMS QTL within identical chromosomal intervals on C2, L and O coincides well with the significantly positive phenotypic correlations among these three traits in phenotypic assay, which provides mutual genetic and phenotypic verification. Differently, BN QTL showed no overlapping location with the above three traits on LG L and formed independent stable loci on LG G. This co-location pattern strongly implies widespread pleiotropic effects or tight gene linkage underlying clustered QTL hotspots.

We further compared our mapped stable loci with previously documented soybean plant architecture QTL resources. Specht et al. [9] reported a plant height QTL adjacent to Satt277 on linkage C2 overlapping with our qPH-C2-1; this conserved interval displayed higher phenotypic contribution (11.7–27.2%) and superior environmental stability across six experimental environments relative to the 5–15% PVE in the earlier research. NNMS QTL on C2 and O in our work fell within published intervals reported by Huang et al. [24]. In contrast, BN QTL on A2 and K from Zhu et al. [26] differed from our Brnach Number-associated genomic regions, reflecting apparent genetic background divergence across mapping populations. Notably, qBPH-O-2 on LG O, stably detected across all six environments with 6.9–31.7% PVE, represents a novel locus for bottom pod height with no prior publication record. Published genetic dissection for BPH remains scarce, making this newly identified QTL valuable for subsequent gene excavation.

Plant architecture traits are influenced by environmental variation. Multi-environment QTL analysis facilitates the identification of environmentally stable QTLs suitable for marker-assisted breeding for molecular breeding. Concentrated QTL hotspots on C2, L and O observed in the present study are not random chromosomal aggregations and may represent conserved genomic regions potentially shaped by long-term soybean domestication. and artificial domestication selection; a similar clustering pattern was previously observed in soybean yield and disease-resistance QTL mapping [22,27]. Co-localized multi-trait QTL clusters originate either from single pleiotropic regulatory genes governing multiple developmental pathways or tight linkage of functionally coupled adjacent genes, two genetic hypotheses requiring further functional validation.

From a breeding perspective, these environment-stable major QTLs, especially the novel qBPH-O-2, can be transformed into user-friendly molecular markers for marker-assisted selection (MAS). Breeders can pyramid favorable alleles from Ji NF58 and Jidou 12 by foreground marker selection to customize compact plant architecture cultivars adapted to dense-planting cultivation in Huanghuai soybean core production zone and shorten traditional phenotypic selection cycles. Within the confidence intervals of three key hotspot regions on C2, L and O, multiple candidate genes annotated in SoyBase participate in gibberellin biosynthesis, auxin signal transduction and stem node development, which are prioritized targets for fine mapping and gene functional verification to resolve the molecular regulatory network of soybean plant architecture. Future fine dissection of these clustered intervals will facilitate efficient favorable gene pyramiding in practical soybean improvement.

## Figures and Tables

**Table 1:** Phenotypic characteristics and broad-sense heritability (H^2^) of plant architecture traits in the RIL population across six environments (two years × three locations).

Trait	Environment		Parents		Max	Min	Average	Range	Kurt.	Skew.	SD	SE	H^2^
	Year	Location	JD12	nf58									
Plant Height	Y1	SJZ	36.93	76.73	93.90	17.53	48.22	76.37	−1.14	0.31	20.01	1.51	78.58
		CZ	81.17	127.00	195.17	30.33	98.68	164.84	−0.08	0.47	32.45	2.45	
		HD	46.67	67.67	118.50	24.00	66.38	94.50	−1.01	0.21	25.10	1.90	
	Y2	SJZ	59.43	99.17	126.92	32.52	72.80	94.40	−0.98	0.16	23.39	1.77	
		CZ	76.60	108.07	149.53	32.67	84.01	116.86	−0.30	0.26	25.75	1.95	
		HD	69.57	121.83	156.00	27.40	84.50	128.60	−0.76	0.14	30.52	2.31	
Bottom Pod Height	Y1	SJZ	11.30	14.13	24.47	5.33	11.56	19.14	0.98	0.83	3.35	0.25	65.50
		CZ	17.67	23.33	42.83	6.00	20.18	36.83	0.83	0.75	6.86	0.52	
		HD	14.17	9.17	38.40	5.67	15.98	32.73	1.21	1.21	6.71	0.51	
	Y2	SJZ	18.40	21.83	35.33	8.53	18.83	26.80	0.71	0.92	5.31	0.40	
		CZ	22.20	22.73	40.00	7.53	19.04	32.47	0.42	0.41	5.97	0.45	
		HD	16.43	23.27	46.17	5.57	17.02	40.60	1.95	1.03	6.67	0.50	
Branch Number	Y1	SJZ	1.87	1.07	4.77	0.27	1.45	4.50	7.86	1.86	0.56	0.04	53.37
		CZ	2.83	0.33	8.00	0.00	2.64	8.00	0.99	0.99	1.51	0.11	
		HD	2.33	2.67	4.17	0.40	2.26	3.77	−0.47	0.10	0.81	0.06	
	Y2	SJZ	1.30	0.97	5.25	0.00	1.67	5.25	1.09	0.80	0.87	0.07	
		CZ	3.13	1.73	6.40	0.47	2.56	5.93	0.16	0.66	1.17	0.09	
		HD	3.13	2.67	4.93	0.20	2.58	4.73	0.06	−0.04	0.87	0.07	
Node Number on Main Stem	Y1	SJZ	11.57	16.30	17.97	6.27	12.25	11.70	−1.37	0.07	3.08	0.23	71.36
		CZ	17.00	19.33	32.50	8.50	17.95	24.00	0.54	0.72	4.56	0.34	
		HD	14.33	16.50	22.50	8.33	14.53	14.17	−0.77	−0.01	3.16	0.24	
	Y2	SJZ	13.17	19.13	21.92	7.80	14.67	14.12	−0.81	−0.03	3.03	0.23	
		CZ	15.93	20.00	24.60	8.53	16.57	16.07	−0.36	−0.10	3.28	0.25	
		HD	14.63	20.83	25.20	8.63	15.90	16.57	−0.75	−0.04	3.62	0.27	

H^2^ (%): Broad-sense heritability.

**Table 2:** Correlation coefficient between different traits in six environment of two years.

Traits		Plant Height		Bottom Pod Height		Branch Number		Node Number on Main Stem	
Name	Location	Y1	Y2	Y1	Y2	Y1	Y2	Y1	Y2
Plant Height	SJZ	1.00	1.00						
	CZ	1.00	1.00						
	HD	1.00	1.00						
Bottom Pod Height	SJZ	0.83 **	0.71 **	1.00	1.00				
	CZ	0.69 **	0.79 **	1.00	1.00				
	HD	0.78 **	0.78 **	1.00	1.00				
Branch Number	SJZ	0.33 **	0.26 *	0.31 **	0.38 **	1.00	1.00		
	CZ	0.44 **	0.40 **	0.25 *	0.31 **	1.00	1.00		
	HD	0.15	0.07	0.05	−0.11	1.00	1.00		
Node Number on Main Stem	SJZ	0.95 **	0.90 **	0.77 **	0.62 **	0.34 **	0.30 **	1.00	1.00
	CZ	0.90 **	0.93 **	0.74 **	0.71 **	0.41 **	0.40 **	1.00	1.00
	HD	0.85 **	0.95 **	0.53 **	0.78 **	0.10	0.04	1.00	1.00

*: Significant at 0.05 level (*p* < 0.05); **: significant at 0.01 level (*p* < 0.01).

**Table 3:** QTLs for plant architecture traits detected by CIM (QTL Cartographer v2.5) across six environments.

Traits	QTL	Environment	Linkage Group	Additive	LOD Score	Position (cM)	Confidence Interval (cM)	Marker Interval	*R*^2^ (%)
PH	*qPH-C2*	E1~E6	C2	(−8.09)~(−14.07)	11.11~18.10	19.9	14.0~20.4	Satt286~Sat_251	15.2~27.2
	*qPH-L*	E1~E6	L	15.00~22.01	18.39~33.12	30.6	28.6~31.2	Satt156~Satt229	33.4~52.5
	*qPH-O-1*	E1~E6	O	7.48~10.79	4.90~6.45	95.1	86.1~104.8	Satt477~Satt592	10.7~13.5
	*qPH-O-2*	E1~E6	O	7.03~10.81	3.64~7.20	115.7	110.8~124.8	Satt581~Sat_190	5.5~14.3
NNMS	*qNNMS-C2*	E1~E6	C2	(−1.22)~(−1.74)	6.33~16.23	17.9	14.0~20.4	Satt286~Sat_251	10.8~23.1
	*qNNMS-L*	E1~E6	L	1.98~2.46	9~27.48	30.6	28.6~31.2	Satt156~Satt229	19.8~52.5
	*qPH-O-1*	E1\E2\E4\E5\E6	O	0.84~1.31	2.83~6.49	95.1	86.1~104.8	Satt477~Satt592	7.1~13.4
	*qPH-O-2*	E1\E2\E5\E6	O	0.98~1.4	3.53~4.36	117.7	111.3~126.3	Satt581~Sat_190	6.4~8.8
BPH	*qBPH-C2*	E1~E6	C2	(−1.79)~(−3.79)	9.23~22.69	17.9	14.0~20.4	Satt286~Sat_251	18~34
	*qBPH-L*	E1\E3\E4\E5\E6	L	1.31~2.98	2.74~10.94	30.6	28.6~31.2	Satt156~Satt229	5.7~20.7
	*qBPH-O-1*	E1~E6	O	1.42~3.01	7.2~11.58	95.1	86.1~104.8	Satt477~Satt592	11.2~30.2
	*qBPH-O-2*	E1~E6	O	1.58~3.08	6.14~15.2	117.7	111.3~126.3	Satt581~Sat_190	11.8~31.7
	*qBN-C2*	E1~E6	C2	(−0.21)~(−0.66)	2.73~13.18	17.9	14.0~20.4	Satt286~Sat_251	6.5~28.3
	*qBN-G*	E1\E3~E6	G	0.17~0.34	2.97~5.53	26	19.1~31.0	Sct_199~Sat_372	8.2~14.7
	*qBN-O-1*	E1/E4	O	0.13~0.21	2.89~2.97	107.1	96.7~108.9	Satt477~Satt592	5.4
	*qBN-O-2*	E1/E4\E5	O	0.16~0.33	2.7~4.59	117.7	110.5~129.0	Satt581~Sat_190	7.6~8.4

E1, Y1 in SJZ; E2, Y1 in CZ; E3, Y1 in HD; E4, Y2 in SJZ; E5, Y2 in CZ; E6, Y2 in HD. PH, plant height; BPH, bottom pod height; BN, branch number; NNMS, node number on main stem; LG, linkage group; LOD, logarithm of odds; PVE, phenotypic variation explained.

**Table 4:** QTL mapping for plant architecture traits in RIL population using MCIM in QTLNetwork2.0.

Traits	Linkage Group	QTL	Marker Interval	F Score	Additive	H^2^_A_(%)	QTL × Environment Interactions						H^2^_AEi_(%)
							AEi1	AEi2	AEi3	AEi4	AEi5	AEi6	
PH	O	*qPH-O-1*	Satt477-Satt592	18.20	6.24	3.50	0.0002	−0.0001	0.0001	0.0001	−0.0001	−0.0001	0.10
	O	*qPH-O-2*	Satt581-Satt190	17.50	5.51	4.00	0.0001	−0.0001		0.0001	−0.0001		0.10
	C2	*qPH-C2-1*	Satt286-Sat_251	56.90	−11.35	11.70	−2.5148	0.7072	−0.2284	−1.0886	1.2520	1.8614	0.40
	J	*qPH-J-1*	Sat_151-Sat_350	5.30	3.17	1.20		0.0001		−0.0001		−0.0001	0.10
	I	*qPH-I-1*	Sat_418-Satt148	6.70	2.21	1.60	0.7001	−2.1627	0.9068	0.2641	0.2415	0.0413	0.20
	L	*qPH-L-1*	Sat_286-Satt229	98.60	15.18	24.80	2.5957	−2.7762	−0.3639	1.0455	2.2169	−2.7386	0.50
	A1	*qPH-A1-1*	Satt236-Sat_271	4.70	−2.66	0.10	−0.1386	1.4523	−0.3890	−0.4026	−0.2837	−0.2457	0.30
	C1	*qPH-C1-1*	Satt578-Satt294	6.80	2.67	1.30	0.0001	−0.0002		0.0002	−0.0001		0.10
BPH	O	*qBPH-O-2*	Satt581-Satt190	30.70	2.51	6.90	0.5427	0.1166	−0.4210	−0.1465	0.0938	−0.1892	0.30
	C2	*qBPH-C2-1*	Satt286-Sat_251	79.60	−3.04	19.60	−1.2629	−0.5547	0.9885	−0.1204	0.3103	0.6509	1.30
	L	*qBPH-L-1*	Satt156-Sat_286	21.20	1.61	8.10	0.2490	0.0303	−0.4166	0.3939	0.2742	−0.5393	0.70
BN	O	*qBN-O-2*	Satt581-Satt190	8.90	0.27	1.20	0.0099	−0.0743	0.0158	−0.0414	−0.0317	0.1231	0.40
	C2	*qBN-C2-1*	Satt286-Sat_251	39.70	−0.45	12.10	−0.1541	0.3116	−0.1891	−0.0578	0.2123	−0.1180	2.80
	G	*qBN-G-1*	Sct_199-Sat-372	9.60	0.27	2.60	0.0019	0.0010		−0.0020	−0.0001	−0.0009	0.30
NNMS	O	*qNNMS-O-1*	Satt477-Satt592	15.70	0.59	3.60		−0.0001	0.0003	0.0001	−0.0001	−0.0002	0.20
	O	*qNNMS-O-2*	Satt581-Sat_190	17.00	0.82	4.10	−0.0001	−0.0002	0.0003	0.0003	−0.0001	−0.0001	0.20
	C2	*qNNMS-C2-1*	Satt286-Sat_251	34.40	−1.36	10.40	−0.0804	0.0152	−0.1093	−0.0578	0.0976	0.1336	0.20
	I	*qNNMS-I-1*	Sat_418-Satt148	7.30	0.46	1.60	0.0703	−0.4963	0.2405	0.0842	0.0617	0.0373	0.50
	L	*qNNMS-L-1*	Sat_286-Satt229	72.10	2.10	24.80	0.0001	−0.0002		0.0001	0.0002	−0.0002	0.10
	A1	*qNNMS-A1-1*	Satt236-Sat_271	5.10	−0.20	0.10	−0.0116	0.2368	−0.0651	−0.0565	−0.0189	−0.0820	0.40

PH, plant height; BPH, bottom pod height; BN, branch number; NNMS, node number on main stem. F score: F statistic value for QTL significance. Additive: additive effect of the QTL. H^2^A (%): proportion of phenotypic variation explained by the additive effect of the QTL. H^2^AEi (%): proportion of phenotypic variation explained by QTL × environment interaction effects. AEi1–AEi6: QTL × environment interaction effects in each of the six environments.

## Data Availability

The original contributions presented in this study are included in the article. Further inquiries can be directed to the corresponding authors.
